# Study of Enzymatic Hydrolysis of Fructans from *Agave salmiana* Characterization and Kinetic Assessment

**DOI:** 10.1100/2012/863432

**Published:** 2012-05-02

**Authors:** Christian Michel-Cuello, Imelda Ortiz-Cerda, Lorena Moreno-Vilet, Alicia Grajales-Lagunes, Mario Moscosa-Santillán, Johanne Bonnin, Marco Martín González-Chávez, Miguel Ruiz-Cabrera

**Affiliations:** ^1^Programa Multidisciplinario de Posgrado en Ciencias Ambientales, Universidad Autónoma de San Luis Potosí, Avenida Dr. Manuel Nava No. 6, Zona Universitaria, 78210 San Luis Potosí, SLP, Mexico; ^2^Facultad de Ciencias Químicas, Universidad Autónoma de San Luis Potosí, Avenida Dr. Manuel Nava No. 6, Zona Universitaria, 78210 San Luis Potosí, SLP, Mexico; ^3^Institut de Chimie Organique et Analytique, Université d'Orléans, Rue d'Issoudun, BP 16729, 45067 Orléans Cedex 02, France

## Abstract

Fructans were extracted from *Agave salmiana* juice, characterized and subjected to hydrolysis process using a commercial inulinase preparation acting freely. To compare the performance of the enzymatic preparation, a batch of experiments were also conducted with chicory inulin (reference). Hydrolysis was performed for 6 h at two temperatures (50, 60°C) and two substrate concentrations (40, 60 mg/ml). Hydrolysis process was monitored by measuring the sugars released and residual substrate by HPLC. A mathematical model which describes the kinetics of substrate degradation as well as fructose production was proposed to analyze the hydrolysis assessment. It was found that kinetics were significantly influenced by temperature, substrate concentration, and type of substrate (*P* < 0.01). The extent of substrate hydrolysis varied from 82 to 99%. Hydrolysis product was mainly constituted of fructose, obtaining from 77 to 96.4% of total reducing sugars.

## 1. Introduction

The term fructans is a generic name assigned to polymers of fructose linked by fructose-fructose glycosidic bonds. If polymer is composed by 2 to 10 fructose molecules, these are known as fructooligosaccharides (FOSs), whereas a properly named fructan is a polysaccharide with a degree of polymerization (DP) greater than 10 molecules of fructose in the chain [1, 2]. In the literature, references have been made to five groups of fructans, which are classified according to their structure and type of bond such as inulin, levan, graminan, neoseries levan, and neoseries graminan [1–4]. The inulin-type fructan, extracted from chicory (*Cichorium intybus*), artichoke (*Cynara scolymus*), and dahlia plant tubercles (*Dahlia variabilis*) has been the most commonly compound used in the food industry due to their functional properties as well as their health benefits. They are nondigestible polysaccharides, considered as prebiotics, since they stimulate the growth and activity of beneficial colon bacteria such as *Bifidobacteria* and *Lactobacillus* [5–7]. Also, fructans have been associated to a decrease of glucose level in blood, homeostasis of lipids, mineral availability, and immunomodulatory effects [8, 9]. Moreover, fructans possess several properties as texture modification, gel formation, moisture retention, and food stabilization, and consequently they are frequently employed as fat and sugar substitutes [10–13].

Alternatively, fructans have also been considered for the production of high fructose syrups (HFSs) as well as fructooligosaccharides (FOSs) [14–17]. Recently, HFS has gradually replaced refined sucrose and is used as low-caloric sweetener in foods and beverages [18]. In addition, it has been found that some functional properties of foods such as flavor, color, solubility, crystallization inhibition are improved when high-fructose syrups are used [19–21].

At industrial scale, fructose syrups are produced by continuous isomerization of glucose obtained from corn starch. During this industrial process, at least two enzymatic steps with very different reaction conditions are involved. Corn starch is hydrolyzed to maltodextrins by alpha-amylase, then saccharified to glucose by glucoamylase and finally isomerized to fructose by glucose isomerase [22–24]. Due to thermodynamical equilibrium, the product is a syrup containing only about 42% fructose (HFS-42) which is used to produce HFS at different commercial levels in fructose such as HFS-55 and HFS-90 [18, 25, 26]. Among these, HFS-55 has been considered as the standard syrup because it has a similar sweetness to the sucrose obtained from cane sugar [22]. However, the process to enhance the fructose content in syrups is costly and thus makes this method uneconomical [27]. Furthermore, the high corn demand for bioethanol production and the increase in its price stimulate to look for alternative starch sources for the fructose syrups production [26, 28].

Concerning the *Agave *plants, these possess fructans, as their main photosynthetic product, synthesized and stored in the stem. These fructans are used by the plants as a source of energy and as an osmoprotector during drought and cold stress periods [29, 30]. In the particular case of the *Agave tequilana*, over 60% of the soluble carbohydrates represents a complex mixture, mainly composed of highly ramified fructans and neofructans [31–33]. At present, the main use of the fructans from *Agave* is to get fermentable sugars for the manufacturing of alcoholic drinks such as tequila, mescal, and sotol [33–36]. Therefore, some authors such as García-Aguirre et al. [17] have regarded the *Agave *plants as a promising source for fructose syrup production due to its high fructan content. Consequently, in Mexico several distilleries have implemented the same cooking step used in tequila or mezcal elaboration process to obtain fructose syrups. To hydrolyze the fructans, agave heads are cooked in brick ovens for approximately 36 h or cooked in autoclaves for about 12 h. The thermal hydrolysis of fructans in these conditions is not suitable due to undesired degradations (Maillard reaction) and the formation of by-products such as phenolic compounds from lignin which may have a significant impact on the flavor and color of these products [33–35]. Hence, enzymatic hydrolysis based on the use of inulinases constitutes a promissory alternative approach for the production of fructose syrup from agaves. Inulinases are *β*-fructan fructanohydrolases produced mainly by bacteria, fungi, and yeast. The use of exoinulinase (EC 3.2.1.80) and the endoinulinase (EC 3.2.1.7) acting either alone or combined, have been widely investigated for partial or total inulin hydrolysis [14–16]. In the particular case of agave fructans, a relatively limited investigation had been carried out so far. For example, a commercial enzymatic preparation (Fructozyme L) with endo- and exoinulinase activities was used by Avila-Fernandez et al. [34] to replace the thermal hydrolysis step and by Waleckx et al. [37] to substitute the conventional chemical treatment applied after cooking step, in both cases to hydrolyse the agave fructans during tequila production. On the other hand, García-Aguirre et al. [17] proposed *Kluyveromyces marxianus*, an endogenous strain isolated from *aguamiel* with capacity for inulinase synthesis which was applied to obtain fructose rich syrups from agave fructans. Generally, the procedure involving enzymatic hydrolysis appears to be very attractive. However, some kinetic aspects of the enzymatic process as well as the assessment of process parameters such as temperature, substrate concentration, and by-product production on the activity and stability of the enzyme need more special attention before their industrial application.

Therefore, the objective of the present study was to investigate the effect of temperature and substrate concentration on the hydrolysis kinetics of fructans extracted from the *Agave salmiana* juice subjected to a commercial inulinase preparation (Fructozyme L) acting freely. Because of the lack concerning data on *Agave salmiana* hydrolysis, it was decided to compare the performance of the commercial enzymatic preparation on a batch of experiments conducted with inulin (standard grade).

## 2. Materials and Methods

### 2.1. Obtention of the Agave Fructan Powder

The *Agave salmiana* plants used were from seven to nine years old, with approximately one year of castration, and collected at Ejido de Zaragoza de Solís, a municipality of Villa de Guadalupe, S L P. in the spring of 2010. The Agave juice was extracted using mill and expeller equipment from the local maguey processing plant located on the same Ejido land. This juice was filtered several times to eliminate the fibers and obtain samples with high-fructan content and convenient for spray drying. The first and second filtrations were carried out by means of a stainless steel press filter (Shriv, 405 Type), using bleached cellulose filter paper with a pore diameter of 22 *μ*m and 4 *μ*m and mixed with 1% of diatomaceous material (Celite) as a filtering aid. The third filtration was performed under vacuum conditions, passing the juice through a Whatman no. 42 filter paper with a pore diameter of 2.5 *μ*m. To inactivate the saponins present in the juice, these were treated with heat at 80°C for 30 min in a water bath with continuous agitation [38]. One batch of homogenized juice was obtained which was immediately dried with a Büchi mini spray dryer (Model B-290, Flawil, Switzerland). The atomizer pressure, the feed rate and the inlet air temperature were kept at 1.5 bar, 6.0 mL/min and 170°C, respectively. Carrier agent was not used. The dried samples were subjected to quantitative and qualitative determination of sugars and used to measure the average-degree polymerization (DP) of agave fructan as described later. These powders were also used as substrate during the hydrolysis experiments.

### 2.2. DP Characterization of Agave Fructan

The DP of the Agave fructan was determinated by the technique of Matrix-Assisted Laser Desorption/Ionization Time-Of-Flight Mass Spectrometry (MALDI-TOF-MS), which has been reported as the best choice to establish the DP distribution of these types of carbohydrates. Experiments were performed in positive ion mode using an AutoFlex I mass spectrometer (Bruker Daltonics). The instrument was operated at an accelerating voltage of 20 kV in linear mode and 19 kV in reflectron mode. The pressure was 1.5 × 10^−7 ^mbar. The sample was ionized with a nitrogen laser (*λ* = 337 nm). The sample was dissolved in water, and the matrix was 2,5-Dihydroxybenzoic acid (10 mg/mL), prepared in 1 : 1 methanol : water. Samples (0.5 *μ*L) and matrix solution were spotted and air dried on a stainless-steel plate. Spectra were acquired in linear mode (200–300 laser shots) in the m/z range from 800 to 10,000 and reflectron mode (150–200 laser shots) in the m/z range from 300 to 2,000. A pepmix calibration kit (Bruker Daltonics) and apomyoglobin were used for calibration.

### 2.3. Carbohydrate Characterization of Agave Fructan and Chicory Inulin

The dried samples were diluted accordingly for sugars determination (glucose, fructose, sucrose and fructans). This was carried out according to the HPLC-method proposed by Zuleta and Sambucetti [39] with a Waters 600 chromatography equipment (Milford MA. USA), consisting of a degassing device, quaternary pump, column thermocompartment, and a refractive-index detector (Waters 410). An Aminex HPX-87C column ion exchange (7.8 mm d.i. × 300 mm, Bio-Rad Hercules, CA, USA) was used as stationary phase. HPLC grade water with a flow of 0.6 mL/min was used as the mobile phase. The volume of the injected sample was 50 *μ*l (with the injector completely full). The column temperature was kept at 75°C. Environmental temperature was kept constant at 20°C. The samples were filtered through nylon-membrane filters (0.45 *μ*m) coupled to 5 mL polypropylene syringes, both from Waters (Milford, CT), and analyzed immediately.

The Quick Start Empower 5.0 was used for system control and data analysis. References sugars (arabinose, fructose, galactose, glucose, lactose, maltose, mannose, ribose, sucrose, and xylose) as well as the chicory inulin (reference compound) were purchased from Sigma-Aldrich. The chromatographic separation time was 20 min and the carbohydrates present in samples were identified by comparing the retention times with those of references sugars. Quantification of the carbohydrates was performed as a functions of the calibration curves, derived for sugars between 0.1–3.2% w/v (*r* = 0.9996) and for fructans between 0.2–8.0% w/v (*r* = 0.9999). The minimum detection level was 0.068 mg/mL. Correlation coefficients (*r*) were calculated between refraction values in samples and standards for each carbohydrate to estimate the detector consistency in terms of concentration amplitude. All the determinations were carried out in duplicate, and a mixture of standards sugars was injected daily on order to identify any calibration variations.

### 2.4. Solid-Phase Microextraction (SPME) of Volatiles Compounds

For the identification of volatile compounds present in samples, SPME technique was used (SPME device from Supelco Inc. Bellefonte, PA, USA). The fiber used was 60 *μ*m diameter and 1 cm length, polyethylene glycol (PEG) coated. For sampling, the SPME fiber was inserted directly into a 20 mL vial with a valve cap with silicone septa containing 100 mg of fructan sample. After that, the sample was heated to 90°C during 5 min before the extraction. Then, the fiber was maintained 10 min at 90°C. The desorption of analytes was performed by heating the fiber in the injection port of the GC-MS equipment at 250°C for 5 seconds (split mode, split ratio 10 : 1).

### 2.5. Analysis of Volatile Compounds by GC/MS

GC-MS analysis was performed using an Agilent HP series 6890 N gas chromatograph (Waldbronn, Germany), coupled to a mass selective detector 5973. A Stabilwax capillary column (30 m × 0.25 mm i.d., 0.25 *μ*m film thickness; Restek) was used for separation. The column temperature program was as follows: 50°C hold 3 min, 3°C/min up to 250°C hold 5 min, splitless injection at 250°C. The detector was operated in electron impact mode. The spectra were collected at 71 eV ionization voltage and analyzed mass range was 15–600 m/z. The transfer line temperature was 250°C and the carrier gas helium flow rate was 1 mL/min. The compounds were identified by comparing their mass spectra with mass spectra library NIST 02.L and by comparison of linear retention indices, relative to a mixture of n-alkanes C_10_–C_28_. All experiments were repeated two times.

### 2.6. Enzyme

The commercial enzyme preparation, Fructozyme L (Novozymes A/S, Danmark), from *Aspergillus niger,* was purchased from Sigma-Aldrich. This enzyme is a mixture of exoinulinase (EC 3.2.1.80) and endoinulinase (EC 3.2.1.7) with inulinase unit of 2000 INU/g recommended to hydrolyze the linkage of inulin. One inulinase unit is the quantity of enzyme that produces 1 *μ*mol of reducing sugar (calculated as glucose) per minute under the reaction conditions used in Novo Nordisk's standard assay procedure. Protein concentration of Fructozyme L was quantified by the Lowry method [40] and a value of 6.75 ± 0.02 mg/mL was found. The experimental procedure of the inulinase assay with chicory inulin as substrate and described in detail by Ortiz-Cerda [41] was used to select the best reaction parameters such as temperature, pH, enzyme dosage, and substrate concentration.

### 2.7. Hydrolysis of Agave Fructan and Chicory Inulin

The enzymatic hydrolysis experiments were carried out in a 125 mL screw-cap Erlenmeyer flask placed in a large water bath with agitation and controlled temperature (Model PB-1400, Boekel Grant Scientific, USA). Preliminary tests allowed to establish a full factorial design considering two types of substrates (chicory inulin and agave fructan), two levels of substrate concentration (40 and 60 mg/mL), and two different temperatures (50 and 60°C). One replicate for each experimental condition was used and a total of 16 runs were conducted (Table 1). 0.0074 mL of fructozyme L was added to 50 mL of substrate solution which indicated that a protein concentration of 0.001 mg/mL was used in all the experiments. The pH of substrate solution was kept at 4.7. The hydrolysis process was monitored by measuring the sugars such as fructose, glucose, and residual fructan by the HPLC method mentioned previously. Therefore, samples of 3 mL of the reaction media were taken periodically and immediately placed in a water bath at 90°C during 1 min and later quenched in ice for 5 min to inactivate the enzymes [20, 37]. Then, samples were filtered in Whatman No. 42 filter paper with a pore diameter of 2.5 *μ*m and afterward passed through 0.45 *μ*m nylon membrane filter to eliminate the enzymes and obtain samples convenient for sugar analysis.

### 2.8. Mathematical Modeling of the Hydrolysis Kinetics

The model proposed by Rica et al. [42] which describes the complete hydrolysis of the substrate on a single stage was used to describe the rate of substrate consumption (inulin and agave fructan) and the rate of fructose production:

(1) $$S_{n}+\left(n-1\right){\text{H}}_{2}\text{O}\overset{\text{Fructozyme}\;\text{L}}{\to }\left(n-1\right)F+G,$$

where *S*, *F*, *G,* are, respectively, substrate, fructose, glucose; and *n* the degree of polymerization. By applying a mass balance to the hydrolysis process and expressing the concentration of each species in terms of mass of compound per unit volume, the kinetics of the process can be expressed by (2):

(2) $$\left(-\frac{dS}{dt}\right)+\left(-\frac{dW}{dt}\right)=\frac{dF}{dt}+\frac{dG}{dt},$$

where *dW*/*dt* expresses the consumption of water involved in the hydrolysis reaction. The model assumes that there exists a stoichiometric relationship between the molar rate of water consumption and the molar rate of fructose production, according to (3):

(3) $$-\frac{dn_{W}}{dt}=\frac{dn_{F}}{dt},$$

where *n*_*W*_ and *n*_*F*_ express molar concentrations of water and fructose, respectively. Considering the molecular weight of water (MW_W_) and fructose (MW_F_), consumption of water in the reaction can be expressed as,

(4) $$-\frac{dW}{dt}=\frac{{\text{MW}}_{W}}{{\text{MW}}_{F}}\frac{dF}{dt}.$$

Thus (2) becomes,

(5) $$\left(-\frac{dS}{dt}\right)=\left(1-\frac{{\text{MW}}_{W}}{{\text{MW}}_{F}}\right)\frac{dF}{dt}+\frac{dG}{dt}.$$

The model most often applied to describe the consumption of substrate in enzymatic reactions is the Michaelis-Menten equation (6):

(6) $$-r_{s}=\left(-\frac{dS}{dt}\right)=\frac{V_{\max}S}{K_{m}+S}.$$

In this study, preliminary tests suggest that experimental conditions used present a typical unsaturated enzyme behavior. Hence, the initial rate does not depend on enzyme activity but is directly proportional to the concentration of the substrate. Therefore, the Michaelis-Menten model becomes a first order kinetics, as described by (7):

(7) $$-r_{s}≅\frac{V_{\max}S}{K_{m}}=kS\;.$$

Applying integral method, constant kinetics *k* can be calculated from experimental results. Moreover, mass balance can be used to estimate product formation.

From the proposed model, the hydrolysis products are fructose and glucose. However, in the case of inulin, the HPLC analysis shows that the formation of glucose is negligible due to its structure (Figure 4(a)). Then, fructose production rate can be directly calculated from substrate consumption rate.

In contrast, for the agave fructan, analysis shows a significant release of glucose concentration as a function of time (Figure 4(b)). Therefore, the relative fractions of fructose and glucose, obtained by HPLC, were used to calculate the evolution of the products concentration.

As mentioned before, for each experiment, the value of constant kinetic *k* was obtained (Table 1) using the integral method programmed in Scilab 5.2.1. The coefficient of determination (*R* ^2^) was used as primary criteria to determine the accuracy of the fit between model proposed and experimental data.

### 2.9. Statistical Analysis

Analysis of variance (ANOVA) was performed with a confidence level of 99% (*P* < 0.01) with Modde 7.0 (Umetric AB) statistical package. A linear mathematical model with interactions was used to analyze the effect of temperature (*T*), substrate concentration (*C*) and substrate type (*S*) on the rate constants *k* values:

(8) $$y=\beta_{0}+\beta_{1}T+\beta_{2}C+\beta_{3}S+\beta_{4}T*C+\beta_{5}T*S+\beta_{6}C*S,$$

where *β*_*i*_  (*i* = 0,1,…, 6) represents the regression coefficients of the model.

## 3. Results and Discussion

### 3.1. DP Profile of Agave Fructan

**Figure 1:**
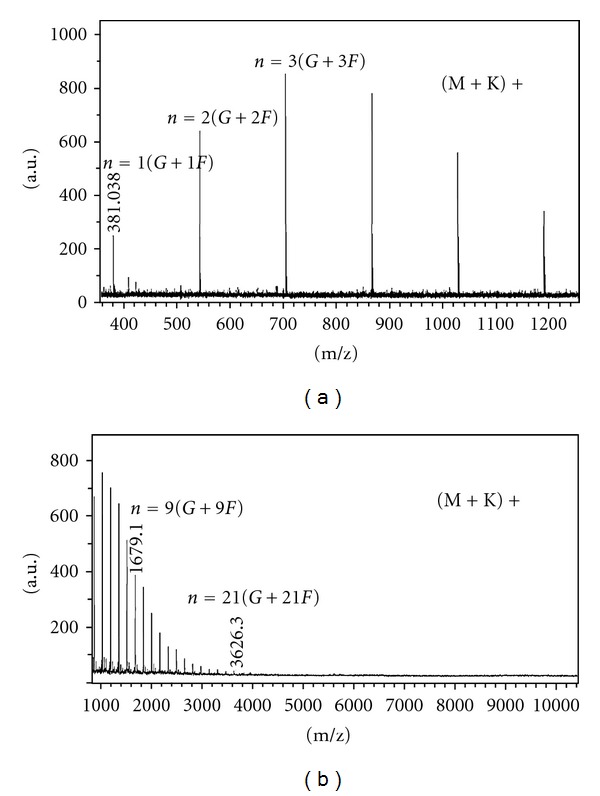
MALDI-TOF-MS spectra, in positive-ion mode of *Agave salmiana* fructan. (a) Low-mass spectra (DP from 1 to 6), (b) high-mass spectra (DP from 4 to 21).

The spectra for the low mass fructan content and high-mass fructan content determined as [M + K]+ ions are shown in Figure 1. It can clearly be seen that the molecular mass distribution of Agave fructan comprised oligomers and polymers of molecular weight ranging from 342.8 to 3587.2 Da, which corresponds to a range of DP from 1 to 21. Nevertheless, the DP average was 8, which was calculated based on the intensity of the peaks of the spectra of high mass fructan content (DP from 4 to 21), considering only the number of fructose units. This is the first report of the DP of fructans from *A.salmiana.*

Diverse studies of molecular structure of the fructan from Agave species, more specific *A. tequilana*, have reported that fructan are based on 1-kestose, neokestose, and branched tetrasaccharides, presenting linkages *β*-(2-1) and *β*-(2-6) between the fructose and some glucose intermediated forming a branched structure [31, 32]. This suggests that fructan from *A. salmiana* might be a similar structure because is a plant of the same species. Also, it could represent important differences in the process of hydrolysis with respect of fructan type inuline from chicory.

### 3.2. Carbohydrates Profiles of Substrates

**Figure 2:**
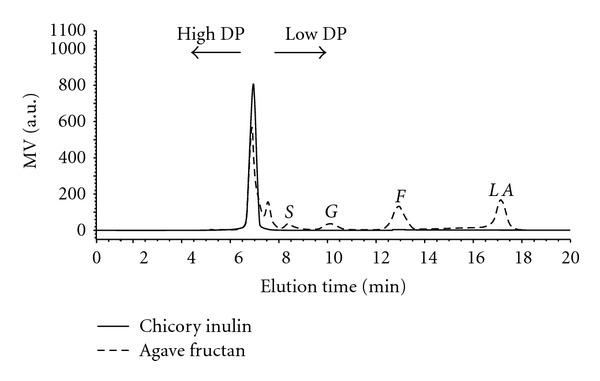
HPLC separation of sugars from agave fructan and chicory inulin used as substrates. *S*: sucrose, *G*: glucose, *F*: fructose, *LA*: lactic acid.

Figure 2 shows the HPLC chromatogram obtained during separation of the different sugars present in the standard chicory inulin and in the fructan extracted from the agave juice. The results showed that fructan and fructose constituted 97% and 3% (dry matter), respectively, of the total sugars present en in the chicory inulin, exhibiting only two peaks with an elution time of 6.6 min and 12.7 min, respectively. Therefore, the chicory inulin from Sigma, used as standard and as substrate in hydrolysis experiments in this work, can be considered as a pure substrate. On the other hand, the average DP of chicory inulin was not indicated by the manufacturer, but it has been reported in literature that commercial inulin are standardized to have an average DP between 12 and 25 [3, 43].

It was also observed that Aminex HPX-87C column separated all the sugars present in the agave fructan powder with a good resolution in a single run of 20 min (Figure 2). Peak separation was good, allowing all peaks to be identified and quantified. Sucrose, glucose, and fructose were identified with elution times of 8.1, 9.8, and 12.7 min, respectively. The main peak of this chromatogram coincides with the peak and elution time (6.7 min) corresponding to the chicory inulin used as a standard. A minor peak with an elution time of 7.5 min was detected just before the sucrose peak which could be considered as a fructan of low DP or fructooligosaccharide. However, for practical considerations regarding this work, these two peaks were quantified as total fructans. It was found in this way that fructan, sucrose, glucose, and fructose constituted 85.6 ± 2.52%, 4.67 ± 0.22%, 3.99 ± 0.14%, and 6.36 ± 0.54%, (dry matter) respectively, of the total sugars present in the *agave* fructan powder. Foregoing, it is evident that agave fructan was partially purified and additional experiments are required using more efficient filtration and purifying process than those used in the present research for treating the *Agave salmiana* juice and obtaining a fructan powder with chemical composition similar to the reference inulin.

**Figure 3:**
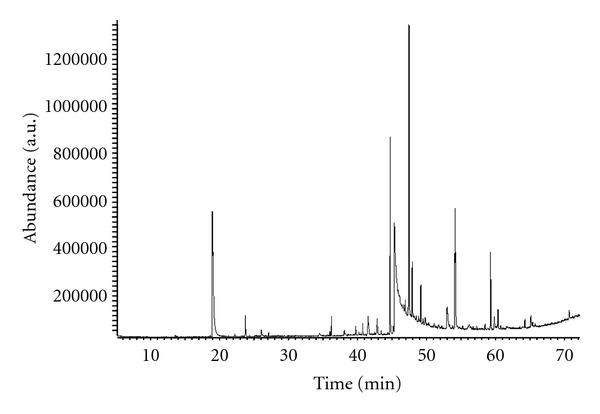
Gas chromatogram of polar volatile compounds of powder fructan obtained with PEG fiber and Stabilwax capillary column.

**Figure 4:**
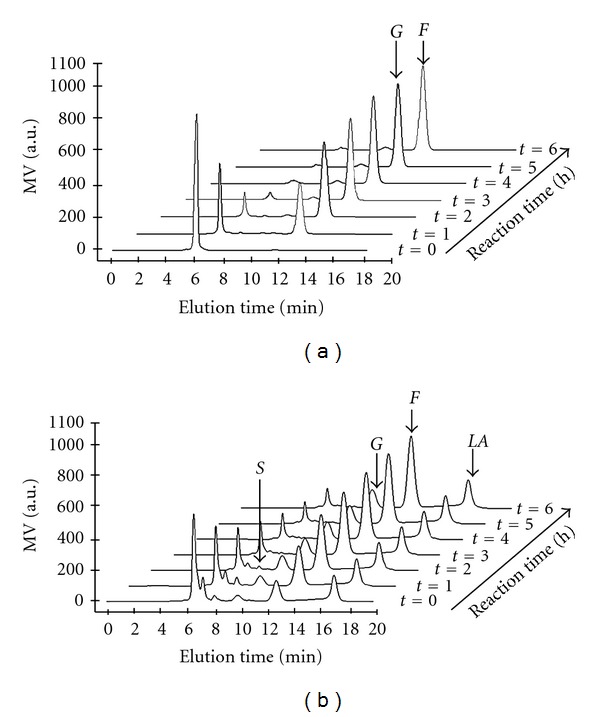
Chromatographic representation of substrates degradation and release of sugars during the enzymatic hydrolysis. (a) Chicory inulin, (b) agave fructan. *S*: sucrose, *G*: glucose, *F*: fructose, *LA*: lactic acid.

The results from the HPLC analysis of samples taken indicate that the kinetics where fructan is used as a substrate, not only are the sugar of interest (Figure 4) but also show the presence of a peak with a retention time (RT) of 17.1 min, which not corresponds with the RT of sugars standards provided. The compound of RT of 17.1 is not a product of the enzymatic hydrolysis of fructan powder, since its concentration remains constant during this process, the tendency of elution in HPLC of fructan goes from fructose polymer to simple sugars (high-to-low-molecular weight, as shown in Figure 3), so we concluded that the peak of 17.1 could be a polar compound and volatile.

The extraction of volatile polar compounds in fructan was performed by SPME and subsequently identified by GC-MS, using both procedures polar fiber and column (Figure 3). The compounds identified by GC-MS are shown in Table 2. The compounds correspond to higher percentage of acetic acid and lactic acid, the retention times obtained by HPLC for both acids are acetic acid 13.4 min and 17.1 min for lactic acid. According to this result, we can say that the peak retention time 17.1 min fructan powder corresponds to lactic acid, this was confirmed by powder fructan enriched with lactic acid. The lactic acid is present in samples with high sugar content as a result of fermentation processes [44, 45].

### 3.3. HPLC Analysis of the Hydrolysis Kinetics

As an example, Figures (4(a) and 4(b)) present the chromatographic evolution of substrate hydrolysis and the corresponding release of sugars for experiments 2 and 6 (Table 1) with chicory inulin and agave fructan as substrates, respectively. In both cases, the reactions were carried out at temperature of 60°C with a substrate concentration of 40 mg/mL. As shown in Figure 4, the degradation of substrates occurred progressively and after 6 h of hydrolysis, fructose was the major product. Figure 4(b) illustrates that when agave fructan was used, the glucose content also increased as a result of fructan and sucrose hydrolysis. The extent of substrate hydrolysis (%) was defined as the ratio of consumed substrate/amount of substrate provided ×100. In this way, at the end of the enzymatic process, values of 92% and 98% were calculated for agave fructan (experiment 6) and for pure inulin (experiment 2), respectively. This disparity in hydrolysis efficiency could be explained by a difference in substrates materials, as chemical composition, impurities presence, and probably due to average DP. However, the range of the percent hydrolysis achieved in this study are analogous to those reported for thermal hydrolysis of fructan contained in *Agave tequilana* [33].

### 3.4. Hydrolysis Kinetics

**Figure 5:**
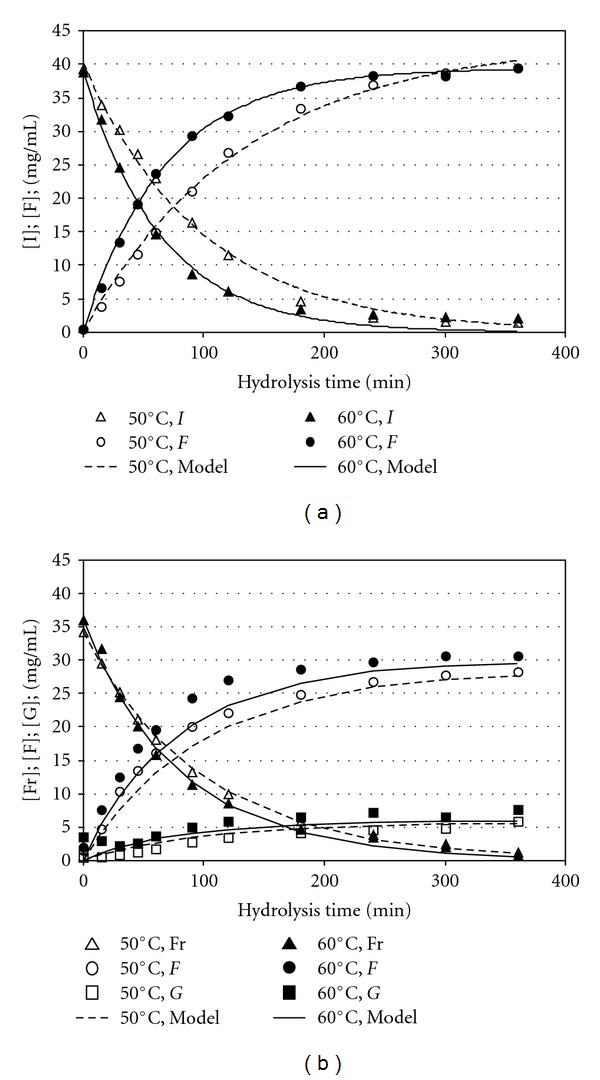
Kinetics of substrates degradation and sugars released as a function of hydrolysis time at different temperatures. (a) Chicory inulin, (b) Agave fructan. *I*: chicory inulin; Fr: agave fructan; *F*: fructose, *G*: glucose.

The experimental and simulated kinetics of substrates degradation as well as sugars release as a function of hydrolysis time, when a substrate concentration of 40 mg/mL of chicory inulin and agave fructan wasused, are shown in Figures 5(a) and 5(b), respectively. Initially, the rate of substrates hydrolysis and the rate of fructose production were very high, and after 200 min, both processes tend, to remain constant. As a general trend it was observed that as temperature was increased, the % hydrolysis and the amount of fructose increased. Therefore, after 360 min of hydrolysis, the maximum production rate of fructose of 39.5 ± 0.02 mg/mL was observed at temperature of 60°C with inulin as substrate (Figure 5(a)). In this case, standard inulin was hydrolyzed about 99.5% and the results of HPLC revealed that the effluent contained 96.4% fructose and 3.4% constituted of glucose and residual inulin. Although so high values of percent hydrolysis (92–95%) were reached when agave fructan was used as substrate (Figure 5(b)), the maximum amount of fructose obtained in the reaction mixture varied between 27.7 and 30.4 mg/mL at 50 and 60°C, respectively. In this case, fructose constituted between 75 and 77% of total reducing sugars in the reaction product. This is due to the accumulation of considerable amount of glucose during hydrolysis process whose proportion increased from 13.00% to 22.37% of total carbohydrates. A similar increase of glucose content was also observed by Waleckx et al. [33] with thermal hydrolysis of fructans from *Agave tequilana*.

**Figure 6:**
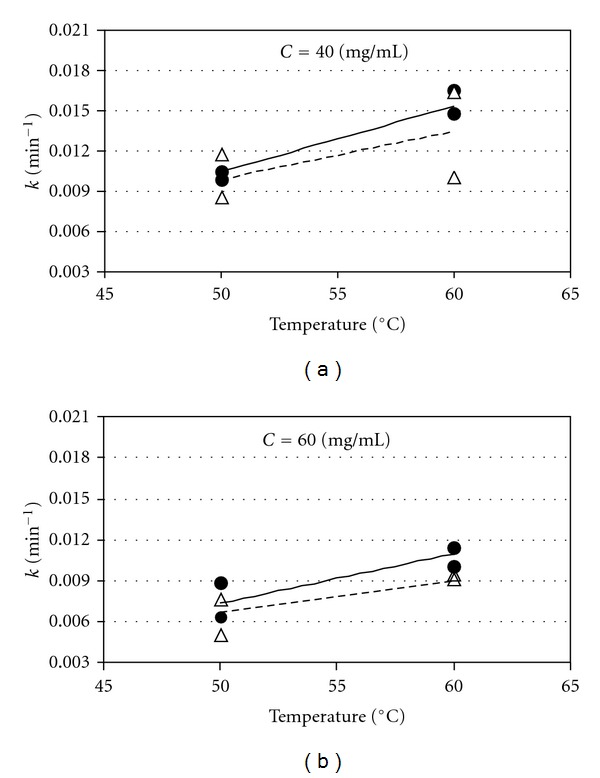
Variation of the rate constant *k* with temperature (a,b). • Chicory inulin; Δ Agave fructan.

The simulated curves are also shown in Figure 5. When calculating the values for rate constants (*k*), the corresponding values for the determination coefficients (*R^2^*) were all greater than 0.978 (Table 1), indicating a good agreement between the experimental and predicted values. An analysis of variance (ANOVA) with a confidence level of 99% (*P* < 0.01) revealed that *k* were affected by the temperature (*T*), the substrate concentration (*C*), and the type of substrate (*S*). It is important to point out that not significant interaction effect between factors was detected in both cases. The identified values of the rate constants *k* as a function of temperature can be observed in Figure 6; the corresponding predicted values of *k* can be also observed.

An analysis of Figure 6 demonstrates that the higher temperatures and smaller substrate concentration resulted in higher values of *k* and consequently faster hydrolysis of substrates. These observations are in agreement with those obtained by Catana et al. [46] in their experiments conducted with inulin as substrate where the optimal temperature for Fructozyme L was around 60°C. Although the obtained values of *k* were smaller when a substrate concentration of 60 mg/mL was used, results concerning % hydrolysis (>92%) in Figure 5 indicated that the amount of enzyme employed (0.001 mg/mL) was enough to hydrolyze almost totally the substrate in 6 h, with no evidence of substrate inhibition. Figure 6 also shows that the experiments conducted with chicory inulin, independent of temperature or substrate concentration, rendered the highest *k* values, resulting in significantly higher hydrolysis rates for inulin. This could be explained by a difference in the structure of substrates. According to literature, inulin is linear fructans consisting mainly of *β*-(2-1) fructosyl-fructose links [1–4], while the fructans of agave plants have been considered as a complex mixture of highly branched fructans presenting the two types of linkages *β*-(2-1) and *β*-(2-6) between fructose moieties [31, 32].

 The Arrhenius equation gives the dependence of the temperature in rate constant *k* in chemical reactions, but it is important to note that this study did not apply the Arrhenius equation because only two values were used as the temperature interval between these is relatively short.

It is clear that the fructose production rate increased as the temperature increased and also it falls as the substrate concentration was raised. This last was due to that the same amount of enzyme was used for both substrate concentrations (40 and 60 mg/mL). The experiments conducted with agave fructan, independent of temperature or substrate concentration, resulted in significantly higher fructose production rates from raw agave fructan. This could be explained because agave fructan shows polymers having low DP and branched structure having more external fructose molecules which can be more easily hydrolyzed by exo-inulinases.

According to the results obtained in this work, the enzymatic process with Fructozyme L might thus be of industrial interest for the large-scale production of high fructose syrup from *Agave salmiana* under appropriate conditions of temperature, enzyme dosage, and substrate concentration. The *Agave salmiana* has been a resource of a wide distribution in the Zacatecan-Potosino *altiplano* (high plateau) which has become a promising raw material for the industrial production of fructose syrup, as well as for the production of fructooligosaccharides and/or fructans.

## Figures and Tables

**Table 1:** Values of the rate constants (*k*) and their respective *R^2^* determined through regression method for each experimental condition.

Exp No.	Run Order	Temperature (°C)	Substrate concentration (mg/ml)	Substrate type	*S_o_* (mg/ml)	*k *(min^−1^)	*R^2^*
1	16	50	40	I	41.1	0.0105	0.991
2	2	60	40	I	39.1	0.0165	0.978
3	15	50	60	I	61.3	0.0064	0.996
4	5	60	60	I	57.9	0.0114	0.996
5	12	50	40	Fr	33.7	0.0086	0.997
6	4	60	40	Fr	40.1	0.0100	0.992
7	6	50	60	Fr	50.4	0.0077	0.995
8	9	60	60	Fr	50.7	0.0091	0.997
9	7	50	40	I	39.5	0.0099	0.997
10	11	60	40	I	39.3	0.0148	0.994
11	8	50	60	I	59.1	0.0089	0.998
12	1	60	60	I	57.2	0.0100	0.997
13	10	50	40	Fr	34.5	0.0118	0.998
14	14	60	40	Fr	32.8	0.0164	0.996
15	13	50	60	Fr	47.9	0.0051	0.998
16	3	60	60	Fr	64.8	0.0095	0.997

I: chicory inulin; Fr: agave fructan.

**Table 2:** Composition of volatile compounds from solid *Agave salmiana*, obtained from the PEG fiber and Stabilwax column (carbowax).

No	Compound	%
1	Acetic acid	15.83
2	2,3-Butanediol	1.00
3	Benzeneacetaldehyde	0.41
4	2-Furanmethanol	0.16
5	Phenethyl alcohol	0.17
6	2,6-di-tert-butyl-p-cresol	0.79
7	2-Acetylpyrrole	0.26
8	2-Formylpyrrole	0.40
9	Caprylic acid	1.79
10	Pelargic acid	7.46
11	Lactic acid	27.49
12	2,3-dihydro-3,5-dihydroxy-6-methyl-4H-Pyran-4-one	14.86
13	Capric acid	2.97
14	2,4-di-tert-butylphenol	5.52
15	Lauric acid	10.06
16	Butyl phthalate	3.53
17	Unidentified	7.3
